# Supplementary material for: Deep Brain Stimulation for Parkinson’s disease changes perception in the Rubber Hand Illusion
Source: Sci Rep. 2018 Sep 14;8:13842. doi: 10.1038/s41598-018-31867-8 (PMC6138647; doi:10.1038/s41598-018-31867-8)

Supplementary Material

TITLE: Deep Brain Stimulation for Parkinson’s disease changes perception in the Rubber Hand Illusion

AUTHORS: Catherine Ding MBBS, FRACP^1^, Colin J Palmer PhD^2,3^, Jakob Hohwy PhD^2^, George J Youssef PhD^4^, Bryan Paton PhD^2,5^, Naotsugu Tsuchiya PhD^6^, Julie C Stout PhD^6^ and Dominic Thyagarajan MD, FRACP^1^

Supplementary Table A: Pre and post Subthalamic deep brain stimulation electrode implantation screening tests in subjects with Parkinson’s Disease

| Test | Pre-operative Median (IQR) | Post-operative Median (IQR) | Statistical test |
| --- | --- | --- | --- |
| Montreal Cognitive Assessment | 27 (25.75-28.25) | 28 (27-29) | *V* = 45.5, *p* = .469 |
| Hospital Anxiety and Depression Scale | 8 (5.75-13.25) | 8 (5-12.75) | *V* = 20, *p* = .637 |
| Apathy Scale | 7.5 (4.75-9) | 10 (5.75-18) | *V* = 10, *p* = .154 |

Supplementary Table B: Rubber Hand Illusion questionnaire

The critical ‘illusion’ items are in **BOLD**. The remaining eight statements were ‘mock’ items

1. **It seemed as though the touch I felt was caused by the paintbrush I could see touching the rubber hand.**
2. **It felt as if the rubber hand was my hand.**
3. It felt as if my (real) hand was turning ‘rubbery’.
4. I found the touch of the paintbrush on my hand was pleasant.
5. It seemed as if I might have more than one right* hand or arm.
6. I found myself liking the rubber hand.
7. **It seemed as if I was feeling the touch of the paintbrush in the location where I saw the rubber hand being touched.**
8. It felt as if my (real) hand was getting cold.
9. I felt the room temperature change during the experiment.
10. The rubber hand began to resemble my own (real) hand, in terms of shape, skin tone, freckles or some other visual feature.
11. It seemed as if I was in two different locations at the same time

* Questionnaire for trials of right-hand state ‘right’ and questionnaire for trials of left-hand state ‘left’.

Supplementary Table C: Evaluation of covariates for questionnaire data (all subjects)


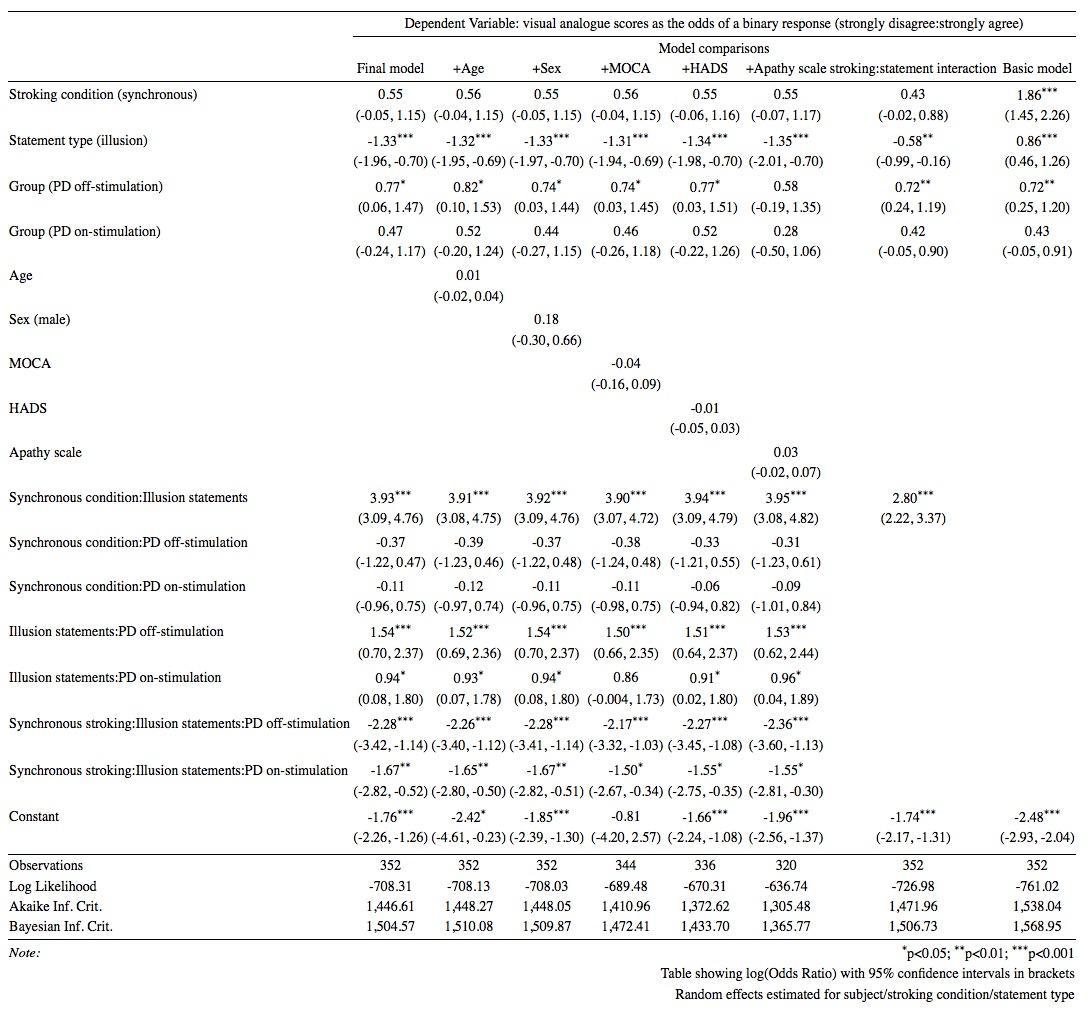


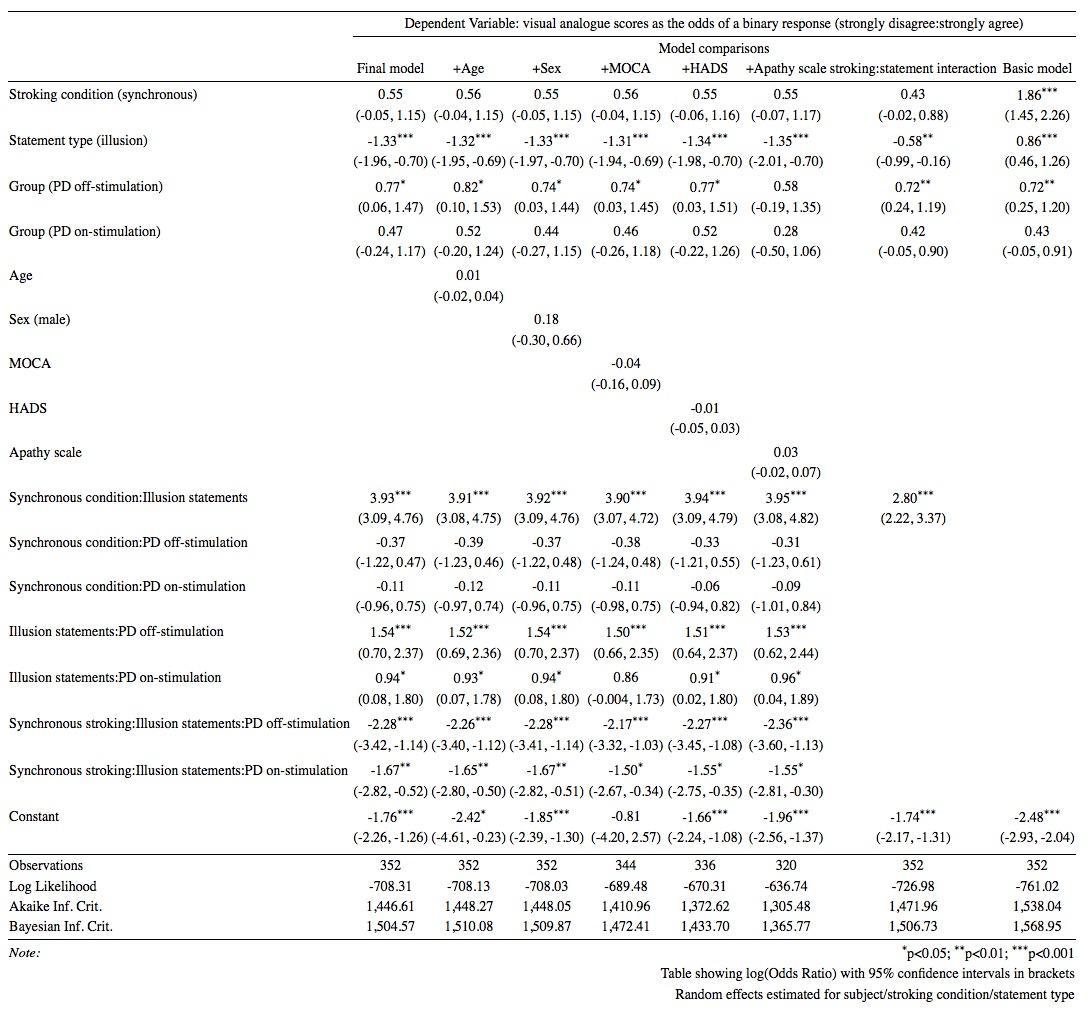


Supplementary Table D: Evaluation of covariates for questionnaire data (subset of preoperative patients)


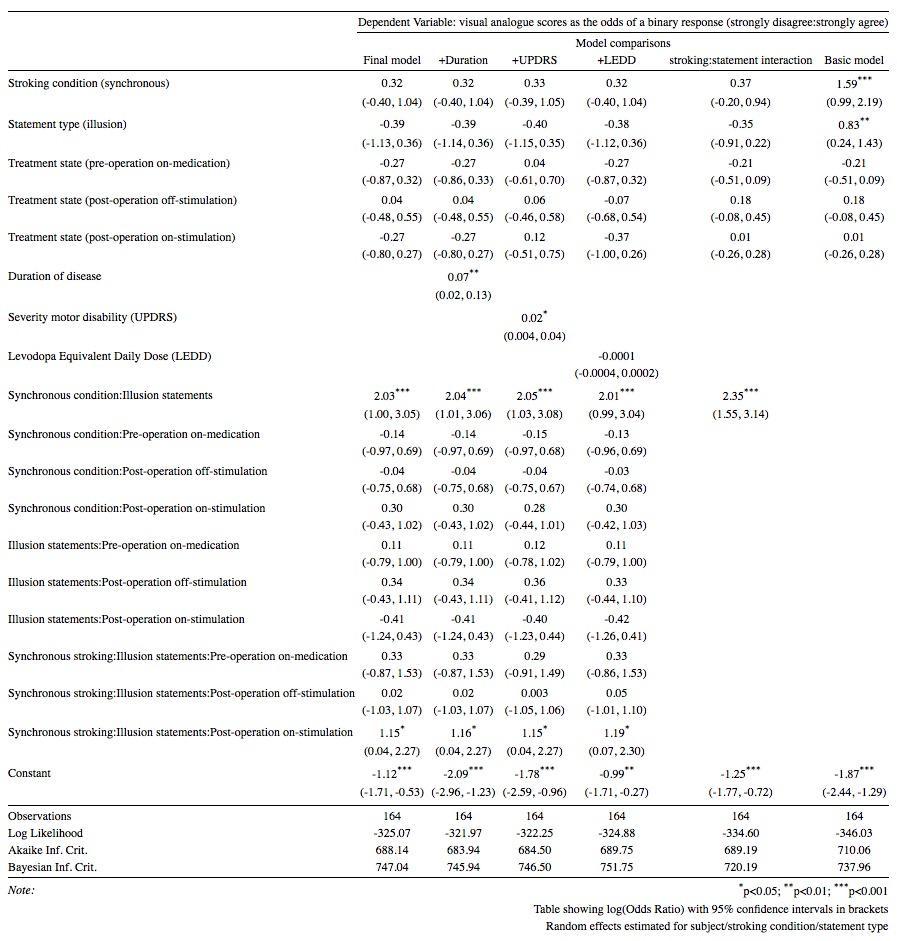


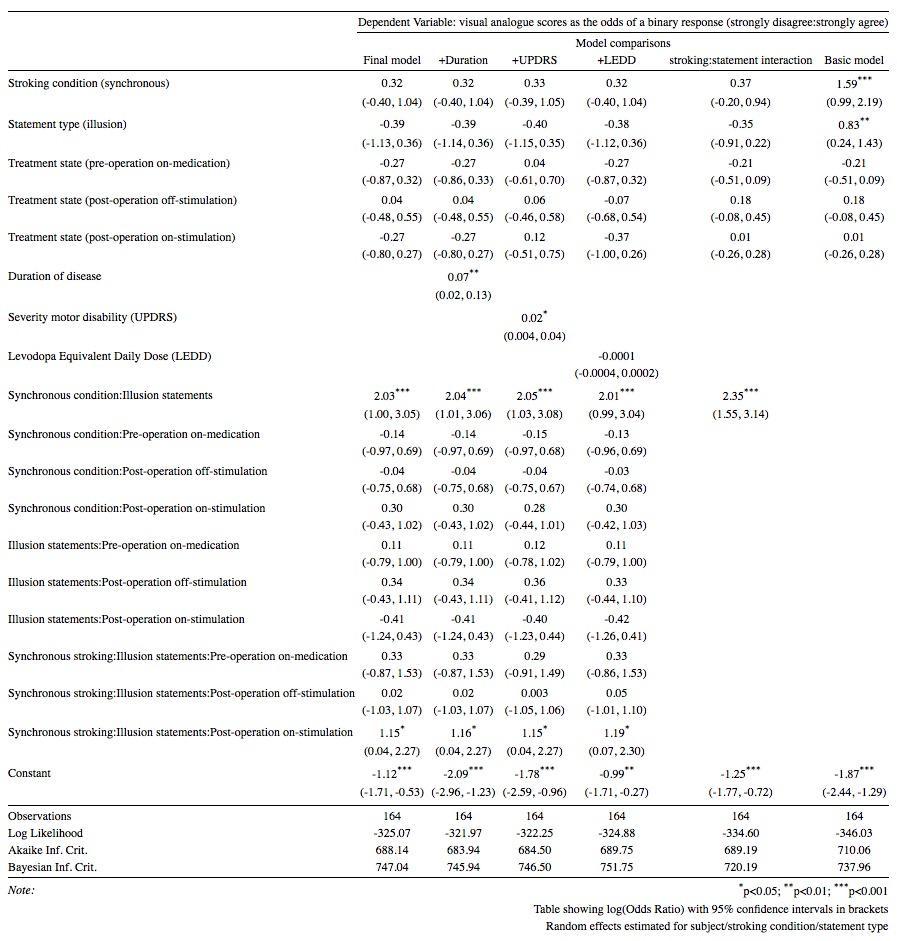


Supplementary Table E: Evaluation of covariates for proprioceptive drift data (all subjects)


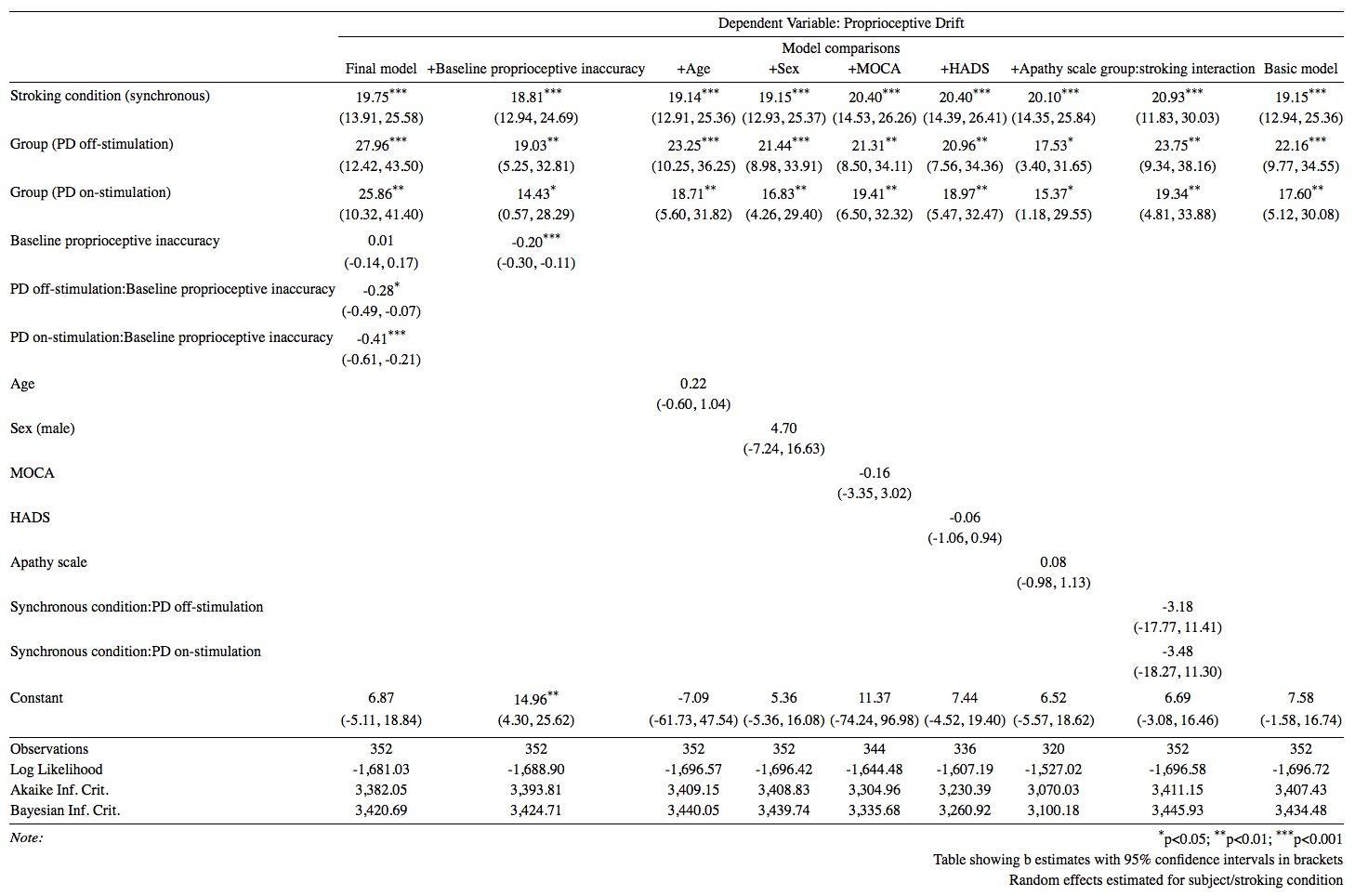


Supplementary Table F: Evaluation of covariates for proprioceptive drift data (subset of preoperative patients)


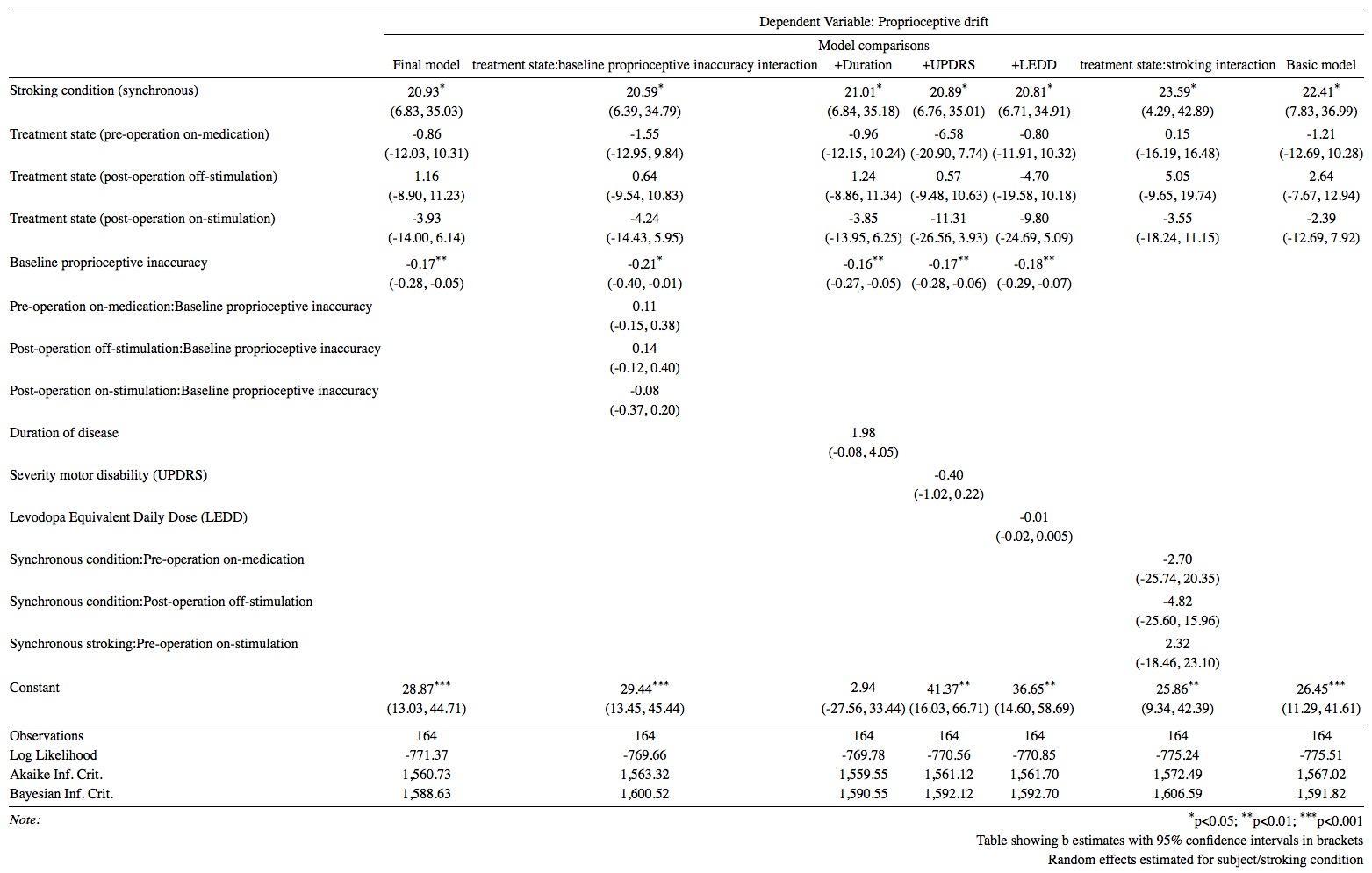


Supplementary Table G: Maximum lateral displacement reach regression models (all Parkinson's disease patients)


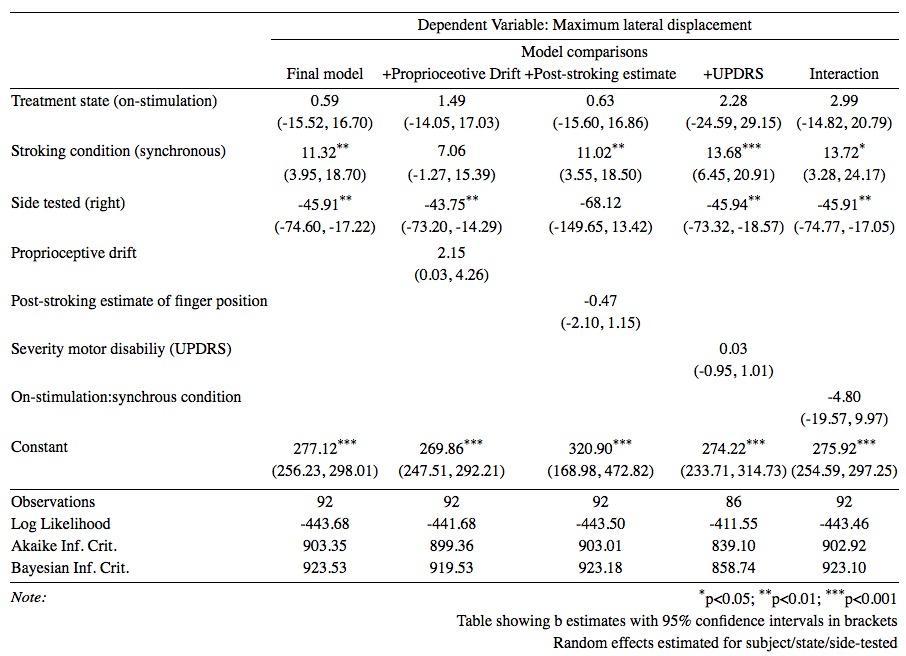


Supplementary Table H: Mean velocity reach regression models (all Parkinson's disease patients)


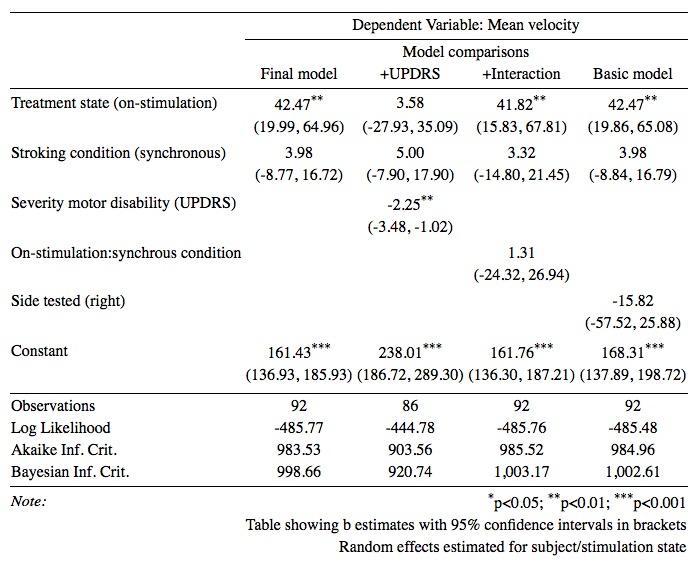


Supplementary Table I: Mean duration reach regression models (all Parkinson's disease patients)


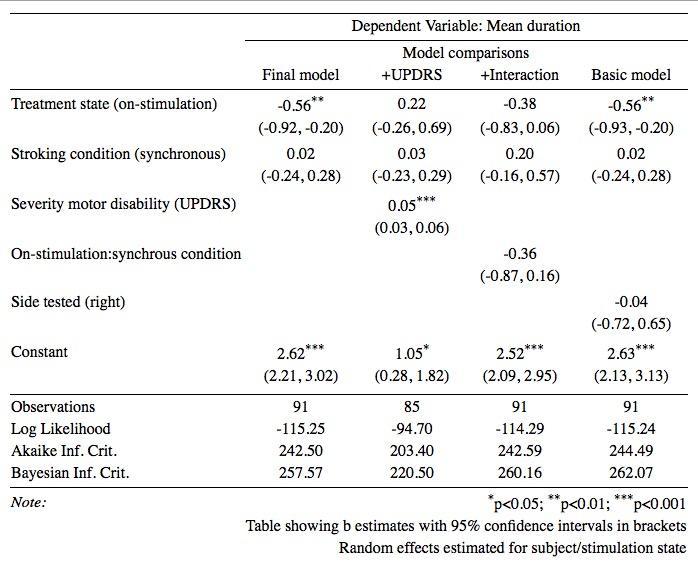


Supplementary Table J: Regression models of remaining reach movement metrics (all Parkinson's disease patients)


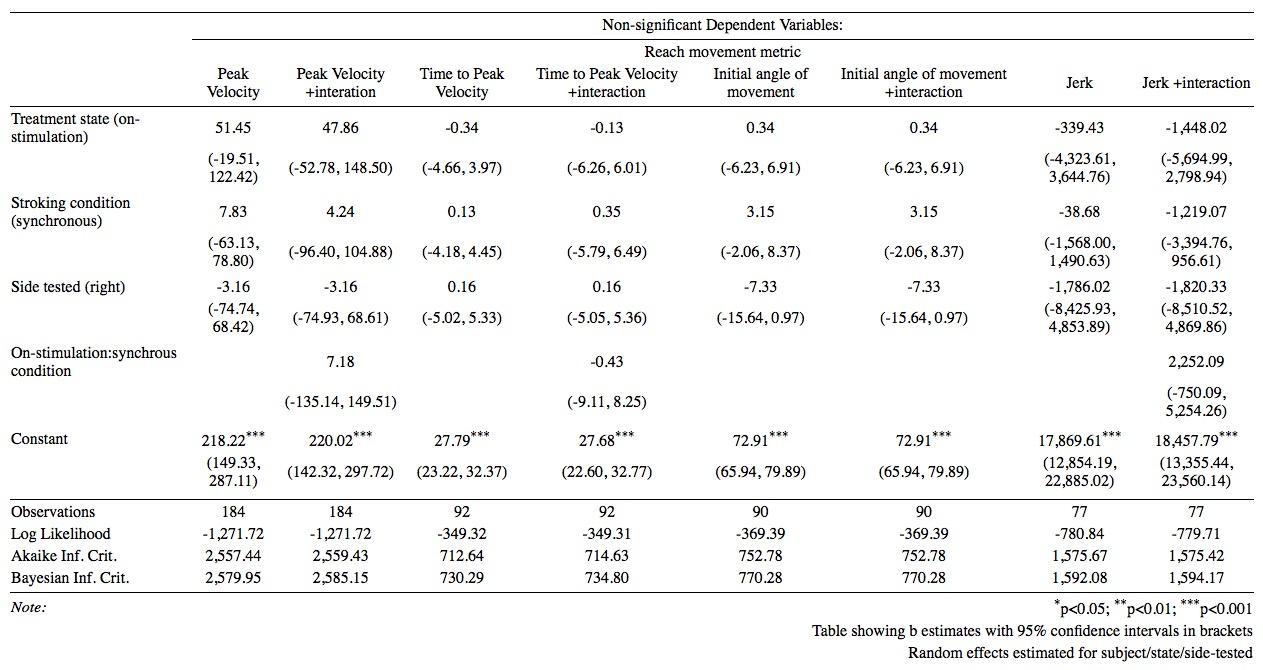

Supplement: Supplementary file 1 — Supplementary Materials [file 41598_2018_31867_MOESM1_ESM.docx]
